# The harmonizome: a collection of processed datasets gathered to serve and mine knowledge about genes and proteins

**DOI:** 10.1093/database/baw100

**Published:** 2016-07-02

**Authors:** Andrew D. Rouillard, Gregory W. Gundersen, Nicolas F. Fernandez, Zichen Wang, Caroline D. Monteiro, Michael G. McDermott, Avi Ma’ayan

**Affiliations:** Department of Pharmacology and Systems Therapeutics, Department of Genetics and Genomic Sciences, BD2K-LINCS Data Coordination and Integration Center (DCIC), Mount Sinai’s Knowledge Management Center for Illuminating the Druggable Genome (KMC-IDG), Icahn School of Medicine at Mount Sinai, New York, NY, USA

## Abstract

Genomics, epigenomics, transcriptomics, proteomics and metabolomics efforts rapidly generate a plethora of data on the activity and levels of biomolecules within mammalian cells. At the same time, curation projects that organize knowledge from the biomedical literature into online databases are expanding. Hence, there is a wealth of information about genes, proteins and their associations, with an urgent need for data integration to achieve better knowledge extraction and data reuse. For this purpose, we developed the Harmonizome: a collection of processed datasets gathered to serve and mine knowledge about genes and proteins from over 70 major online resources. We extracted, abstracted and organized data into ∼72 million functional associations between genes/proteins and their attributes. Such attributes could be physical relationships with other biomolecules, expression in cell lines and tissues, genetic associations with knockout mouse or human phenotypes, or changes in expression after drug treatment. We stored these associations in a relational database along with rich metadata for the genes/proteins, their attributes and the original resources. The freely available Harmonizome web portal provides a graphical user interface, a web service and a mobile app for querying, browsing and downloading all of the collected data. To demonstrate the utility of the Harmonizome, we computed and visualized gene–gene and attribute–attribute similarity networks, and through unsupervised clustering, identified many unexpected relationships by combining pairs of datasets such as the association between kinase perturbations and disease signatures. We also applied supervised machine learning methods to predict novel substrates for kinases, endogenous ligands for G-protein coupled receptors, mouse phenotypes for knockout genes, and classified unannotated transmembrane proteins for likelihood of being ion channels. The Harmonizome is a comprehensive resource of knowledge about genes and proteins, and as such, it enables researchers to discover novel relationships between biological entities, as well as form novel data-driven hypotheses for experimental validation.

Database URL: http://amp.pharm.mssm.edu/Harmonizome.

## Introduction

Currently, biomolecular data are stored in many disjoint online databases. The data within these databases is structured, and thus suitable for data integration; however, most attempts to integrate knowledge from multiple resources have only succeeded in accomplishing this for a few resources. For example, web-based platforms such as BioGPS (1), NCBI’s Entrez Gene Database (2), UniProt (3), GeneWeaver (4), MSigDB (5), GO-Elite (6) or Ingenuity Target Explorer, provide knowledge about genes from the Gene Ontology (GO) (7), protein domains, protein–protein interactions, expression in tissues, membership in pathways, and literature references but there are many other sources that these sites are missing. The knowledge that is commonly missing includes, e.g. gene-phenotype associations, putative regulation of genes by transcription factors, membership of proteins in complexes, putative regulation of genes by microRNAs, and changes in expression after drug treatment, or changes in expression in disease, or after single gene perturbations such as knockdown, knockout, mutation or over-expression. GeneCards (8) is becoming one of the most comprehensive resources for collective knowledge about genes and proteins, aggregating information from over 120 resources. However, GeneCards is a commercial product that does not provide the data through an open and free application programming interface (API). GeneCards is advertising commercial products such as antibodies, compounds, recombinant proteins, and gene sequencing services. This limits the utility of GeneCards for integrative knowledge discovery and pure data mining. Another leading resource is UniProt (9). UniProt focuses on sequence information and employs careful manually curated protein pages with less emphasis on data from omics resources. Other resources use text-mining strategies to collect information about genes and proteins. For example, resources such as WikiGenes (10), iHOP (11), Genes2Wordcloud (12) and EvidenceFinder (http://labs.europepmc.org/evf) identify and highlight genes and other semantic entities in sentences from abstracts and full-text publications to summarize gene and protein functions. These resources suffer from literature research focus biases (13); the uneven attention researchers give to well-studied genes and proteins (14).

One of the challenges related to integrating knowledge about genes and proteins is the standardization of data formats and harmonizing identifiers (15). Integration efforts made in subdomain areas such as protein–protein interactions have already developed successful solutions (16, 17). These solutions require some level of abstraction (15, 18, 19), i.e. ignoring quantitative details specific to a data resource (20, 21). Here we demonstrate that such an abstraction approach is feasible for integrating data about genes and proteins from many online resources. Using a simple schema, such data integration effort directly translates to a useful web service and a gateway to knowledge discovery with many applications (Figure S1D).

## Results

### Datasets and data resources

To create the Harmonizome, we collected information about human and mouse, genes and proteins, from 125 unique datasets (Tables 1–9, Supplementary Table S1) hosted by 72 open online resources (Supplementary Table S2). The collected datasets cover six broad categories of information about mammalian genes or proteins: (i) disease and phenotype associations, (ii) genomic profiles, (iii) physical interactions, (iv) proteomic profiles, (v) structural or functional annotations and (vi) transcriptomic profiles (Supplementary Figure S1A). The datasets provide evidence for associations between genes/proteins and biological entities spanning nine broad categories (Supplementary Table S3 and Supplementary Figure S1B), whereas the evidence types supporting the gene-entity associations span five broad categories (Supplementary Table S4 and Figure S1C). Half of the datasets are from high-throughput, data-driven studies, a third are from low-throughput, hypothesis-driven studies, and the remainder are from mixed sources.

**Table 1:** Datasets. List of datasets group by attribute, with dataset citations

Dataset	Citations
Achilles Cell Line Gene Essentiality Profiles	(22–24)
BioGPS Cell Line Gene Expression Profiles	(1, 25, 26)
CCLE Cell Line Gene CNV Profiles	(27)
CCLE Cell Line Gene Expression Profiles	(27)
CCLE Cell Line Gene Mutation Profiles	(27)
COSMIC Cell Line Gene CNV Profiles	(28, 29)
COSMIC Cell Line Gene Mutation Profiles	(28, 29)
GDSC Cell Line Gene Expression Profiles	(30)
Heiser et al., PNAS, 2011 Cell Line Gene Expression Profiles	(31)
HPA Cell Line Gene Expression Profiles	(32)
Klijn et al., Nat. Biotechnol., 2015 Cell Line Gene CNV Profiles	(33)
Klijn et al., Nat. Biotechnol., 2015 Cell Line Gene Expression Profiles	(33)
Klijn et al., Nat. Biotechnol., 2015 Cell Line Gene Mutation Profiles	(33)
BioGPS Human Cell Type and Tissue Gene Expression Profiles	(1, 25, 26)
BioGPS Mouse Cell Type and Tissue Gene Expression Profiles	(1, 25, 26)
HPM Cell Type and Tissue Protein Expression Profiles	(34)
ProteomicsDB Cell Type and Tissue Protein Expression Profiles	(35)
Roadmap Epigenomics Cell and Tissue DNA Methylation Profiles	(36, 37)
Roadmap Epigenomics Cell and Tissue Gene Expression Profiles	(36, 37)
Allen Brain Atlas Developing Human Brain Tissue Gene Expression Profiles by Microarray	(38–40)
Allen Brain Atlas Developing Human Brain Tissue Gene Expression Profiles by RNA-seq	(38–40)
GTEx Tissue Sample Gene Expression Profiles	(41, 42)
HPA Tissue Sample Gene Expression Profiles	(32)
TCGA Signatures of DEGs for Tumors	(43)
Allen Brain Atlas Adult Human Brain Tissue Gene Expression Profiles	(37–40, 44)
Allen Brain Atlas Adult Mouse Brain Tissue Gene Expression Profiles	(38, 39, 45)
Allen Brain Atlas Prenatal Human Brain Tissue Gene Expression Profiles	(38–40, 46)
GTEx Tissue Gene Expression Profiles	(41, 42)
HPA Tissue Gene Expression Profiles	(32)
HPA Tissue Protein Expression Profiles	(32)
TISSUES Curated Tissue Protein Expression Evidence Scores	(47)
TISSUES Experimental Tissue Protein Expression Evidence Scores	(47)
TISSUES Text-mining Tissue Protein Expression Evidence Scores	(47)

List of datasets group by attribute, with dataset citations. Datasets providing evidence for associations between genes and ‘cell lines, cell types or tissues’.

**Table 2:** Datasets providing evidence for associations between genes and ‘chemicals’

Dataset	Citations
CTD Gene-Chemical Interactions	(48, 49)
SILAC Phosphoproteomics Signatures of Differentially Phosphorylated Proteins for Drugs	
DrugBank Drug Targets	(50, 51)
Guide to Pharmacology Chemical Ligands of Receptors	(52)
HMDB Metabolites of Enzymes	(53, 54)
CMAP Signatures of DEGs for Small Molecules	(55)
GEO Signatures of DEGs for Small Molecules	(56–58)
LINCS L1000 CMAP Signatures of DEGs for Small Molecules	(59)
KinomeScan Kinase Inhibitor Targets

**Table 3:** Datasets providing evidence for associations between genes and ‘diseases, phenotypes or traits’

Dataset	Citations
GEO Signatures of DEGs for Diseases	(56, 57)
CTD Gene-Disease Associations	(48, 49)
DISEASES Curated Gene-Disease Assocation Evidence Scores	(61)
DISEASES Experimental Gene-Disease Assocation Evidence Scores	(60)
DISEASES Text-mining Gene-Disease Assocation Evidence Scores	(60)
GAD Gene-Disease Associations	(61)
GAD High Level Gene-Disease Associations	(61)
GWASdb SNP-Disease Associations	(62)
PhosphoSitePlus Phosphosite-Disease Associations	(63, 64)
ClinVar SNP-Phenotype Associations	(65)
GWAS Catalog SNP-Phenotype Associations	(66)
GWASdb SNP-Phenotype Associations	(62)
HPO Gene-Disease Associations	(67)
HuGE Navigator Gene-Phenotype Associations	(68)
MPO Gene-Phenotype Associations	(69–72)
OMIM Gene-Disease Associations	(73, 74)
dbGAP Gene-Trait Associations	(75, 76)

**Table 4:** Datasets providing evidence for associations between genes and ‘functional terms, phrases or references’

Dataset	Citations
GO Biological Process Annotations	(7, 77)
GeneRIF Biological Term Annotations	(78)
Phosphosite Textmining Biological Term Annotations	
COMPARTMENTS Curated Protein Localization Evidence Scores	(79)
COMPARTMENTS Experimental Protein Localization Evidence Scores	(79)
COMPARTMENTS Text-mining Protein Localization Evidence Scores	(79)
GO Cellular Component Annotations	(7, 77)
LOCATE Curated Protein Localization Annotations	(80)
LOCATE Predicted Protein Localization Annotations	(80)
GO Molecular Function Annotations	(7, 77)
Biocarta Pathways	
HumanCyc Pathways	(81, 82)
KEGG Pathways	(83, 84)
PANTHER Pathways	(85, 86)
PID Pathways	(87)
Reactome Pathways	(88, 89)
Wikipathways Pathways	(90)
CORUM Protein Complexes	(91, 92)
NURSA Protein Complexes	(93, 94)
ESCAPE Omics Signatures of Genes and Proteins for Stem Cells	(95)
GeneSigDB Published Gene Signatures	(96, 97)

**Table 5:** Datasets providing evidence for associations between genes and ‘other genes, proteins or microRNAs’

Dataset	Citations
MSigDB Cancer Gene Co-expression Modules	(98)
GEO Signatures of DEGs for Gene Perturbations	(56, 57)
LINCS L1000 CMAP Signatures of DEGs for Gene Knockdowns	(59)
MSigDB Signatures of DEGs for Cancer Gene Perturbations	(98)
SILAC Phosphoproteomics Signatures of Differentially Phosphorylated Proteins for Gene Perturbations	
Hub Proteins Protein–Protein Interactions	(99)
BIND Biomolecular Interactions	(100, 101)
BioGRID Protein–Protein Interactions	(102, 103)
DIP Protein–Protein Interactions	(104)
HPRD Protein–Protein Interactions	(105, 106)
IntAct Biomolecular Interactions	(107, 108)
NURSA Protein–Protein Interactions	(93, 94)
Pathway Commons Protein–Protein Interactions	(109)
GEO Signatures of DEGs for Kinase Perturbations	(56, 57)
KEA Substrates of Kinases	(110)
PhosphoSitePlus Substrates of Kinases	(63, 64)
SILAC Phosphoproteomics Signatures of Differentially Phosphorylated Proteins for Protein Ligands	
Guide to Pharmacology Protein Ligands of Receptors	(52)
MiRTarBase microRNA Targets	(111, 112)
TargetScan Predicted Conserved microRNA Targets	(113–115)
TargetScan Predicted Nonconserved microRNA Targets	(113–115)
DEPOD Substrates of Phosphatases	(116)
GEO Signatures of DEGs for Transcription Factor Perturbations	(56, 57)
CHEA Transcription Factor Targets	(117)
ENCODE Transcription Factor Targets	(118, 119)
JASPAR Predicted Transcription Factor Targets	(120, 121)
TRANSFAC Curated Transcription Factor Targets	(122, 123)
TRANSFAC Predicted Transcription Factor Targets	(122, 123)
Virus MINT Protein-Viral Protein Interactions	(124)

**Table 6:** Datasets providing evidence for associations between genes and ‘molecular profiles’

Dataset	Citations
Kinativ Kinase Inhibitor Bioactivity Profiles	
ENCODE Histone Modification Site Profiles	(118, 119)
Roadmap Epigenomics Histone Modification Site Profiles	(36, 37)
CHEA Transcription Factor Binding Site Profiles	(117)
ENCODE Transcription Factor Binding Site Profiles	(118, 119)

**Table 7:** Datasets providing evidence for associations between genes and ‘organisms’

Dataset	Citations
GEO Signatures of DEGs for Viral Infections	(56, 57)
Virus MINT Protein-Virus Interactions	(124)

**Table 8:** Datasets providing evidence for associations between genes and ‘sequence features’

Dataset	Citations
GTEx eQTL	(41, 42)

**Table 9:** Datasets providing evidence for associations between genes and ‘structural features’

Dataset	Citations
InterPro Predicted Protein Domain Annotations	(125–128)

To harmonize the 125 datasets we: (i) organized each incoming dataset into a matrix with genes labeling the rows and biological entities (attributes) labeling the columns; and (ii) standardized identifiers for genes and biological entities; while also (iii) calculated standardized scores for gene-biological entity associations; and (iv) computed gene–gene and entity–entity similarity matrices. These matrices were then: (v) saved to text files and (vi) loaded into a relational database. To manage gene or protein identifiers, we mapped them all to NCBI Entrez Gene Symbols. To consolidate biological entity identifiers we mapped these to existing ontologies for tissues, cell lines, chemicals, functional terms, phenotypes and diseases (Supplementary Table S5). To serve the data in useful formats, we provide all gene–entity-value triplets for download as text files in matrix, gene-set library, biological entity-set library and bi-partite graph formats. In addition, gene–gene and entity–entity similarity networks for each dataset are also available.

### The harmonizome web resource

To accommodate users who seek information about a single gene, as well as computational biologists who can programmatically operate on the data, the Harmonizome includes advanced search functionality, and serves the data in text file and JSON formats through an API. The Harmonizome landing page displays a search bar where users can type in any search term with autocomplete capabilities (Supplementary Figure S2A). The engine searches for matching datasets, genes and attributes. On the search results pages users can choose to view datasets, genes or attributes pages (Supplementary Figure S2B). These pages contain metadata and provide various views. The Harmonizome site also has a global summary visualization of the knowledge about each gene across all of the datasets. This interactive heat map, called the Harmonogram, displays the genes as the rows and the datasets as the columns. The intensity of each square on the Harmonogram indicates the relative number of functional associations that each gene has in each dataset (Supplementary Figure S2C). This visualization reveals gaps in knowledge about genes, and suggests where to focus future experiments to illuminate functions of unannotated genes to increase potential for novel discoveries.

For further visual exploration of the data, the Harmonizome includes interactive heat maps of hierarchically clustered: (i) datasets (gene–biological entity relationships matrices), (ii) gene–gene similarity matrices, (iii) entity–entity similarity matrices and (iv) dataset pairs (matrices comparing biological entities from one dataset to biological entities from another dataset based on similarity of their gene associations).

**Figure 1.**
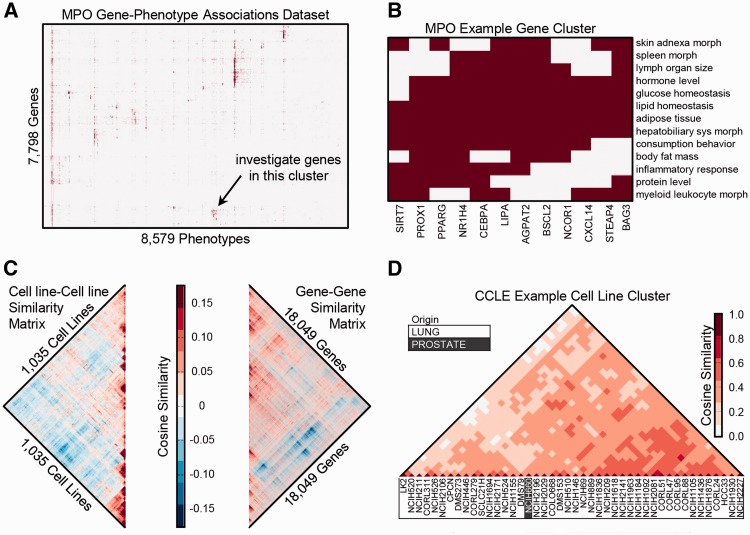
Hierarchical clustering of gene-term, term-term and gene-gene matrices. **(A)** Gene-phenotype associations from the MPO organized into a binary matrix and clustered using hierarchical clustering. **(B)** Zooming into a cluster of genes with similar associated phenotypes, filtered to show higher level phenotypes associated with at least half of the genes in the cluster but no > 10% of all genes. **(C)** The gene–gene and cell-line/cell-line similarity matrices are from the CCLE gene expression dataset. Along the main diagonal of both matrices, there are several distinct zones of high red intensity, indicating clusters of cell lines with similar differentially expressed genes (DEGs) and clusters of genes with similar patterns of expression across cell lines. **(D)** Zooming into the lung cancer cell-lines cluster.

Hierarchically clustered data matrices in the Harmonizome collection can uncover new knowledge. For example, we organized phenotype data from the Mammalian Phenotype Ontology (MPO) (129) into a binary matrix with genes labeling the rows, phenotypes labeling the columns, and matrix elements set equal to 1 to indicate which phenotypes were observed following knockout of a gene. Hierarchical clustering of this matrix shows patches of common phenotypes for groups of genes (Figure 1A). By exploring the clustered heat map visualization of the MPO dataset, we noticed a small group of genes (NCOR1, BAG3, SIRT7, STEAP4, CXCL14, CEBPA, PROX1, AGPAT2, BSCL2, LIPA, NR1H4 and PPARG) that are associated with abnormalities of both the immune system and metabolism, such as glucose homeostasis, lipid homeostasis and feeding behavior (Figure 1B). Interactive hierarchical clustering plots with zooming and panning capabilities are available on the Harmonizome site, enabling further exploration of this type of clustering analysis.

Hierarchically clustered functional association networks (130) can also be explored for each dataset. We derived gene–gene and entity–entity functional association networks by computing the cosine similarity of the rows and columns of each dataset, respectively. In the cancer cell-line encyclopedia (CCLE) dataset, as an example, we can observe correlated gene expression modules and groups of cell lines (Fig. 1C). The cell lines from CCLE predominantly cluster by tissue of origin. However, in a few interesting instances, some cell lines are in clusters of a different tissue; e.g. NCI-H660 is marked as prostate tissue, but appears within a cluster of 43 lung cancer cell-lines (Figure 1D). The ATCC website states that NCI-H660 was originally a small-cell lung carcinoma cell-line, but this cell line was later reclassified to extra-pulmonary lymph node metastatic cancer originating from the prostate (131–133). The cell-line similarity heat map strongly supports a lung origin/phenotype. Interactive gene-gene and attribute–attribute functional association networks with zooming and panning capabilities are available on the Harmonizome site, potentially uncovering many other unexpected relationships.

**Figure 2.**
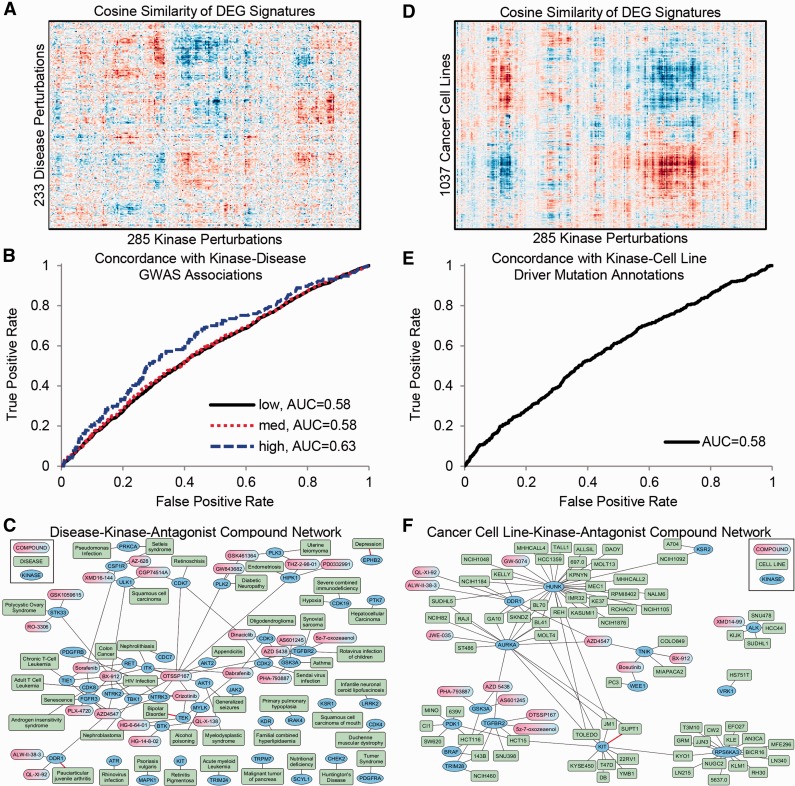
Example of combining datasets: matching kinases with diseases and drugs. **(A)** Hierarchical clustering of kinase perturbation signatures extracted from GEO and disease signatures extracted from GEO. **(B)** Validation of kinase-disease associations with genomics datasets. ROC curve showing concordance of kinase-disease associations derived by comparing gene expression profiles and kinase-disease associations collected from GWAS and other genetic association datasets. Low, medium and high labels correspond to confidence levels of associations from GWAS datasets. **(C)** Network showing top predictions of drug-kinase-disease associations. Red edges indicate kinase-disease associations that have supporting GWAS evidence. **(D)** Hierarchical clustering of signatures of DEGs for kinase perturbations extracted from GEO compared with signatures for cancer cell lines from CCLE. **(E)** ROC curve showing concordance of kinase-cell line associations derived by comparing gene expression profiles and driver kinase mutations for cell lines from COSMIC. **(F)** Network showing top predictions of drug-kinase-cell line associations. Red edges indicate kinase-cell line associations supported by COSMIC as having a driver mutation in the cell line.

Users of the Harmonizome can combine two or more datasets to identify relationships that are only possible to uncover once these datasets have been abstracted, normalized, organized and combined. We devised two related case studies to demonstrate this concept. For the first case study, we integrated differentially expressed gene (DEG) signatures for kinase perturbations with DEG signatures for diseases. The similarity scores for 233 disease signatures paired with 285 kinase perturbation signatures mostly did not match; however, we observed clear patches of positive and negative correlations (Figure 2A). The positive correlations (red patches) suggest that the kinase, or its pathway, is likely perturbed in the disease. The negative correlations (blue patches) suggest diseases in which downregulating the kinase may reverse expression toward the normal tissue expression and promote a more favorable phenotype. Hence, these kinases are potential drug targets for the specific disease. To confirm this conjecture, we found that some of the similarity scores were predictive of kinase-disease associations obtained from genome-wide association studies (GWAS) and other genetic association datasets in the Harmonizome (Figure 2B). Finally, we integrated knowledge about small molecules that inhibit kinases by combining the kinase-disease similarity network with the LINCS KinomeScan dataset to create a tri-partite graph connecting small molecules to kinases to diseases as potential therapeutics (Figure 2C).

In the second case study, we performed a similar analysis, but here we replaced the disease signatures with signatures of DEGs for cancer cell lines from CCLE to derive similarity scores for 1037 cancer cell lines paired with 285 kinase perturbations (Figure 2D). These similarity scores were predictive of driver gene mutations in the cancer cell lines as reported by the COSMIC resource (28) (Figure 2E). Finally, we integrated the LINCS KinomeScan dataset to create a tri-partite graph connecting cancer cell lines to likely driver kinases to kinase-inhibitor compounds (Figure 2F). Experimental methods can assess whether some of these compounds selectively influence the phenotype of these cells. Integration with the recently published cancer cell-line sensitivity data is an alternative (27,30, 31,134). Indeed, some of the predicted small molecules have already been tested and shown to have favorable effects on the cancer cell lines and diseases suggested by our analysis. For example, sorafenib has shown promise for the treatment of colorectal cancer (135); dinaciclib for the treatment of malignant gliomas (136); and bosutinib for melanoma (137), prostate cancer (138) and pancreatic cancer (139). These confirmations suggest that some of our predictions are correct, and some can serve as a global reference point for further analyses to provide other rational and novel hypotheses for experimental validation. These case studies illustrate just two of many ways to combine the Harmonizome datasets for discovery and hypothesis generation. The Harmonizome website provides the ability to explore similar relationships between pairs of datasets by performing unsupervised hierarchical clustering of similarity matrices comparing biological entities between datasets.

### The harmonizome mobile app

The Harmonizome mobile application serves the biological knowledge we collected in an easy-to-access interface where a user can enter a gene of interest to discover properties and functions for the gene (Supplementary Figure S3). Developed using the Facebook React Native platform, the Harmonizome mobile app serves knowledge about genes organized into eight categories, and provides links to external sources for further exploration of gene-function associations. The Harmonizome mobile application is free and available at the Google Play Store (http://goo.gl/JWlI8H) for Android devices, and the App Store (http://appstore.com/harmonizome) for iOS devices. A demonstration video with a case study is available on YouTube at: https://youtu.be/dkYcD51pnfY.

### Machine learning case studies

On its own, the Harmonizome web resource is a valuable tool for discovery and hypothesis generation by enabling exploration of functional associations between mammalian genes and diseases/phenotypes, tissues and other biological entities collected from over a hundred diverse datasets. However, there is also the opportunity for discovering new knowledge about mammalian genes and proteins by the ‘guilt-by-association’ concept, i.e. genes and proteins that share some common functional properties are likely to share more of those properties. To demonstrate this concept we utilized the Harmonizome data for developing four predictive models using Machine Learning. These case studies demonstrate how to use the Harmonizome data for predicting novel properties for genes and proteins.

#### Predicting ion channels from uncharacterized transmembrane proteins

Discovery of novel ion channels could open new lines of research and reveal potential drug targets (140). Ion channels have diverse structures and this makes it challenging to discover ion channels based on sequence information alone. For example, ion channels vary in their number of transmembrane domains and are commonly part of macro-molecular complexes (141). Searching gene or protein sequences for transmembrane domains is useful for predicting proteins that are located in the plasma membrane, but channel activity is much more difficult to predict computationally from sequence alone. Roughly 5500 genes have been predicted to give rise to transmembrane proteins (142). We can use the omics data within the Harmonizome to construct a Machine Learning classifier to predict if any uncharacterized transmembrane proteins are likely ion channels. Our overall modeling approach can be broken down into three stages: (i) gene and dataset selection; (ii) dimensionality reduction and feature selection; and (iii) model training, cross-validation and finally making predictions.

We began with 5428 human genes predicted by Fagerberg *et al.* (142) to encode for transmembrane proteins. We next divided these genes into three classes: 341 known ion channels, 4510 non-ion channels and 577 uncharacterized genes. Next, we selected datasets from the Harmonizome to obtain attributes for the ion channel classifier. We considered only omics datasets, ranked each dataset by the predictive value of its attributes, and retained a final set of 8 datasets covering 320 ion channels (94%), 3928 non-ion channels (87%) and 396 uncharacterized transmembrane genes/proteins (69%).

From each of those eight datasets we next selected the best attributes as predictors for training the ion channel classifier. For this we performed principal component analysis on each of the selected datasets, retained the principal components needed to capture 99% of the variance of each dataset, and then concatenated the principal components from all datasets into a single matrix. This process yielded 6985 total predictors. We performed receiver operating characteristic (ROC) analysis to rank the value of each predictor for discriminating between ion channels and non-ion channels. We used the Breiman Random Forest algorithm with decision trees to train ion channel classifiers and found that 70 features and 300 trees were sufficient to achieve near minimal out-of-bag error. The final set of 70 features contained contributions from all eight datasets, with the majority of the features coming from the InterPro structural domains dataset (Supplementary Table S6).

**Figure 3.**
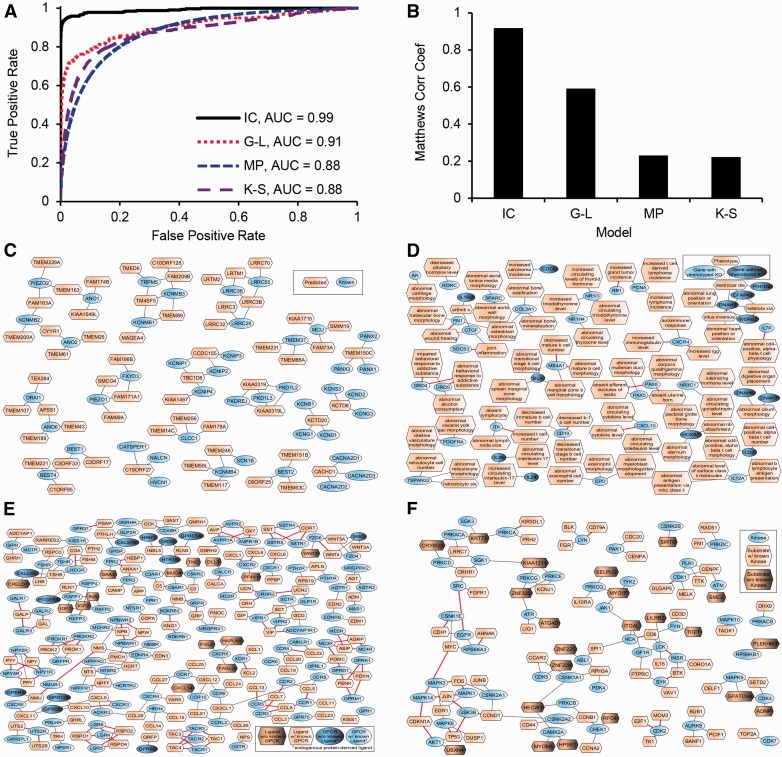
Example of supervised machine learning: classifiers to predict ion channels (IC), phenotypes of single gene knockouts in mice (MP), ligands of GPCRs (G-L), and substrates of kinases (K-S). **(A)** ROC curve of the classifiers. **(B)** MCC as a function of the fraction of correct predictions. **(C)** Network showing candidate ion channels, predicted at a false discovery rate (FDR) of 0.67, connected to their most similar known ion channels, and limited to no more than three edges per node. **(D)** Network showing candidate gene-phenotype associations, predicted at a FDR of 0.33, limited to no more than three edges per node, and trimmed to remove clusters with all edges supported by prior knowledge. Red edges indicate known associations. **(E)** Network showing candidate GPCR-ligand interactions; predicted at a FDR of 0.67 and limited to no more than three edges per node. Red edges indicate known interactions. **(F)** Network showing candidate kinase-substrate interactions predicted at a FDR of 0.67 and limited to no more than three edges per node. Red edges indicate known interactions.

The area under the ROC curve of the final classifier was 0.99 (Figure 3A). The F1 score and Matthew’s Correlation Coefficients (MCC) had maximum values of 0.922 and 0.918 (Figure 3B, Supplementary Figure S4 and Supplementary Table S7). These performance statistics, calculated from the out-of-bag data, estimate how well the classifier generalizes to data not seen while training the model. We used the model to predict and rank ion channel probabilities for the 396 uncharacterized genes (Supplementary Table S8). To provide context for these predictions, we computed a network connecting each predicted ion channel to its most similar known ion channels (Figure 3C). In summary, we can determine with high confidence the molecular function of uncharacterized transmembrane genes/proteins that are likely ion channels and have the potential to become drug targets. The first step for experimentally validating such predictions is to express these genes in artificial systems that can test channel activity.

#### Predicting mouse phenotypes for single gene knockouts

The Mouse Phenotype Ontology (129) currently contains phenotype data for ∼7000 single gene knockouts in mice. Knockout phenotype data are valuable for generating hypotheses about the function, tissue specificity and disease relevance of mammalian genes. The International Mouse Phenotyping Consortium is working toward systematically phenotyping single gene knockouts for the remainder of the genome (143). This is an expensive and time-consuming effort projected to complete in 2021.

In a similar way as described earlier for ion channels, we used omics datasets from the Harmonizome to build a model to predict phenotypes for single gene mouse knockouts. Instead of training a single model to predict a single gene label, i.e. an ion channel, we trained many models to predict many labels (2666 phenotypes). Observed phenotypes of mice harboring single gene knockout mutations obtained from the Mouse Genome Database (71, 129) were the positive training examples, while single gene knockouts with unobserved phenotypes were the negative training examples. The area under the ROC curve of the phenotype classifier was 0.88 (Figure 3A). The F1-score and MCC had maximum values of 0.24 and 0.23 (Figure 3B, Supplementary Figure S5 and Supplementary Table S7). We used the model to predict phenotypes for 7934 single gene mouse knockouts (Supplementary Table S9), and created a gene-phenotype network to visualize a subset of the top predictions (Figure 3D). Our computational predictions of phenotypes for single gene knockouts can assist in prioritizing genes for experimental phenotyping. Furthermore, such predictions, if combined with mouse models of disease, have the potential to identify novel drug-target candidates.

#### Predicting endogenous ligands for G protein-coupled receptors

G-protein-coupled-receptors (GPCRs) are important biologically and pharmacologically due to their roles as sensors and signal transducers (144). GPCRs are the most successful protein family currently serving as targets for drugs; yet most research efforts have focused on relatively few GPCRs (145). At present, there are over 140 orphan GPCRs, which are GPCRs with no known ligand (146). So far, most computational approaches have attempted to predict ligands for GPCRs using structure-based methods. As a complementary method, we used datasets from the Harmonizome to build a classifier to predict protein ligands for GPCRs, although we are aware that GPCRs can bind non-protein ligands. First, we extracted known GPCR-ligand interactions from the Guide to Pharmacology (52). This allowed us to assign GPCR-candidate ligand pairs to positive, negative, or unknown classes for model training and predictions. Using the same procedure as described above for ion channels, the area under the ROC curve of the GPCR-ligand interaction classifier was 0.91 (Figure 3A). The F1-score and MCC had maximum values of 0.59 (Figure 3B, Supplementary Figure S6 and Supplementary Table S7). We used the model to classify 368 953 GPCR-ligand pairs involving either a GPCR with no known endogenous protein ligand, or a candidate ligand with no known GPCR interaction (Supplementary Table S10). Finally, we created a GPCR-ligand network to visualize a subset of the top predictions (Figure 3E). The discovery of endogenous ligands for these GPCRs could open new lines of biological and pharmacological research. Methods that screen ligands for GPCRs rapidly emerge (147, 148) and these predictions can inform such efforts.

#### Predicting substrates of kinases

Protein kinases are well-studied enzymes that regulate almost all cellular processes by reversible phosphorylation of their substrates (149, 150). Kinases are also a promising family of drug targets. While phosphoproteomics studies have revealed many phosphorylation sites, and the human kinome is highly annotated, our knowledge of kinase-substrate interactions remains vastly incomplete. For developing the PhosphoSitePlus database, investigators from Cell Signaling Inc. (151) aggregated information about phosphorylation sites on proteins from low-throughput published studies in the literature, and high-throughput mass spectrometry studies, finding ∼108 000 phosphorylation sites on 12 500 human proteins. We also aggregated information about kinase-substrate phosphorylation reactions from few databases and found about 3500 human proteins with at least one known kinase that phosphorylates them (110). This leaves thousands of proteins with at least one phosphorylation site but with no known upstream regulatory kinase.

To attempt filling this knowledge gap, we used the Harmonizome data collection to build a classifier to predict substrates for kinases. We began with 8293 human proteins with reported phosphorylation sites. We used the kinase enrichment analysis (KEA) dataset (110) to divide these proteins into two classes: 3552 substrates with a known upstream kinase, and 4741 substrates with unknown upstream kinase. Next, we selected datasets from the Harmonizome to build the classifier. We initially considered 34 datasets that cover at least 95% of the substrates. After an initial dataset selection process, we ultimately left with a final set of 12 datasets covering 3,363 substrates with at least one known kinase (95%), and 4270 substrates with unknown kinase (90%). We then performed feature selection using principal component analysis, retaining features that capture 99% of the variance of each dataset. This analysis reduced the number of features to 75. Using a similar scheme as described earlier, we predicted novel kinase–substrate interactions between kinases and substrates with no known kinase. Known kinase–substrate interactions from KEA (110) were used to define positive and negative classes for training the model. The area under the ROC curve of the kinase-substrate interaction classifier was 0.88 (Figure 3A). The F1-score and MCC had maximum values of 0.23 and 0.22 (Figure 3B, Supplementary Figure S7 and Supplementary Table S7). We used the model to classify 2 993  096 potential kinase-substrate pairs involving either a kinase with no known substrate, or a candidate substrate with no known regulatory kinase (Supplementary Table S11). Finally, we created a kinase-substrate network to visualize a subset of the top predictions (Figure 3F). The prediction of kinase-substrate associations is still missing the site of the phosphorylation, the functional effect of the phosphorylation, and the context of the phosphorylation. However, it provides a reliable mapping at a more abstract level, and a resource that can direct experimental testing towards detailed direction of discovery. It can also assist in the reconstruction of the human kinome network, i.e. how kinases regulate each other.

## Discussion

To create the Harmonizome resource, we had to make many decisions in regards to cutoffs for significance of differential expression analysis, data normalization methods, similarity measures between genes and terms, merging IDs for genes and proteins, and combining IDs across mammalian organisms. In addition, in many cases we had to ignore details such as the location of a single nucleotide polymorphism (SNP), the location of a binding site in proximity of a coding region, location of phosphorylation sites on a protein, physical interactions between proteins in a complex and more. This form of data abstraction was necessary for data integration (20, 21). To impute knowledge from observed functional associations between genes and their attributes, we constructed Random Forest classifiers for four supervised Machine Learning tasks. We chose the Random Forest classifier because it is nonlinear, nonparametric, regularized and simple to train (152, 153). To achieve better performance, we could have trained an ensemble of different high-performing classifiers. Furthermore, the performance of the classifiers can be improved in many ways, e.g. by using a multivariate feature selection method. Another limitation of our initial approach may be that the negative class for the training examples was not always purely negative. For example, to predict substrates of kinases, ideally, we would benefit from negative class examples. In practice, the negative class consisted of proteins where it is unknown experimentally whether the kinase phosphorylates the substrate. Regardless of these potential limitations, we believe that our predictions represent a set of credible data-driven hypotheses suitable for experimental validation.

So far, we have noticed that the Harmonizome web service has been highly accessed. Form October 2015 to May 2016, over 33 000 unique users accessed the site. In the near future, we plan to add complex querying capabilities, on the fly Machine Learning, and communities of users centered on a gene or a dataset of interest. In addition, we can organize and serve knowledge about drugs and small molecules in a similar way. Another feature that we plan to implement is providing suggestions for similar genes or drugs to those currently displayed. We plan to continually maintain and expand the Harmonizome while keeping it a free and open resource.

## Methods

### Data processing

We extracted gene- entity-value triplets from each dataset and stored these data in matrices with genes labeling the rows and biological entities labeling the columns. The values in these matrices are discrete or continuous, depending on the data source. We standardized continuous-valued datasets to create more harmonized datasets. Our strategy was to standardize each continuous-valued dataset to have values ranging from 0 to 1, or −1 to 1, where 1 indicates strong positive gene-entity association, –1 indicates strong negative gene-entity association and 0 indicates no observed gene-entity association. Negative values applied to datasets where it was appropriate to convey signed information, e.g. up-regulation and down-regulation for gene expression datasets. To implement this strategy, for each continuous-valued dataset, we converted the values to empirical cumulative probabilities, which transformed the values to range from 0 to 1. If the median values for the genes were different, we computed the probabilities gene-by-gene, otherwise we computed the probabilities on all of the data at once. When appropriate to convey sign information, we doubled the probabilities and subtracted unity, which transformed the values to range from −1 to 1. After creating the standardized datasets, we created binary or tertiary datasets by applying a threshold to retain only 10% of the strongest gene-biological entity associations.

The processing steps to convert each data matrix to a binary or tertiary matrix depends on the data type and processing steps already taken by the original data provider. Any of the following operations may have been part of a data processing pipeline: filtering rows or columns, averaging rows or columns, imputation, transformation/scaling and quantile normalization. Each dataset page on the Harmonizome website provides a script documenting the processing steps used for each dataset. These scripts are also available on GitHub.

### Identifier mapping

For each dataset, we mapped gene or protein identifiers to NCBI Entrez Gene Symbols and Gene IDs for human genes. Overall, we encountered six types of identifiers: NCBI Entrez Gene IDs, gene symbols, Ensembl Gene IDs, UniProt Accessions, genomic coordinates given as nucleotide position(s) on a chromosome and microarray Probeset IDs. We utilized ID mapping tables maintained by NCBI Entrez Gene, Ensembl, UniProt, Hugo Gene Nomenclature Committee (HGNC), Mouse Genome Informatics (MGI) and the Gene Expression Omnibus (GEO) to convert identifiers to NCBI Entrez Gene Symbols and Gene IDs. We used the mapping table maintained by NCBI Homologene to convert mouse NCBI Entrez Gene IDs to human Entrez Gene IDs.

Specifically, for gene symbols, we obtained lists of retired or synonymous gene symbols for NCBI Entrez Gene IDs from NCBI Entrez Gene, HGNC and MGI. From these lists, we created a table mapping gene symbols to NCBI Entrez Gene IDs and official Gene Symbols. We then filtered the table, removing symbols that mapped to more than one NCBI Entrez Gene ID and removing symbols that were identical to official Gene Symbols. For Ensembl Gene IDs, we downloaded tables from Ensembl mapping Ensembl Gene IDs to NCBI Entrez Gene IDs for human and mouse genes. For UniProt Accessions, we downloaded tables from UniProt mapping UniProt Accessions to NCBI Entrez Gene IDs for human and mouse proteins. For genomic coordinates, we downloaded tables from Ensembl listing the chromosome, gene start position, gene end position and transcription start site of each Ensembl Gene ID for human and mouse genes. We joined these tables with the previously described tables mapping Ensembl Gene IDs to NCBI Entrez Gene IDs to derive a table mapping genomic coordinates to NCBI Entrez Gene IDs. For microarray Probeset IDs, we downloaded the platform annotation tables from GEO, mapping Probeset IDs to gene symbols, NCBI Entrez Gene IDs, or Ensembl Gene IDs. We joined these tables with the mapping tables described above to derive tables mapping Probeset IDs to NCBI Entrez Gene IDs. We discarded data for unconverted gene or protein identifiers. We documented the original identifier, number of identifiers and fraction of unmapped identifiers (Supplementary Table S12). The median fraction of unmapped identifiers was 3%. Many of the unmapped identifiers correspond to predicted genes and other forms of untranslated to protein non-coding genes.

We mapped labels for tissues, cell lines, chemicals, functional terms, phenotypes and diseases to terms in relevant ontologies and dictionaries, which we refer to as naming authorities. If we matched a label to a term or synonym from one naming authority, we linked the original label to that term and its metadata including name, description, identifier and persistent URL. Otherwise, we did not change the original label.

### Harmonizome web resource implementation

The Harmonizome web server is a Java servlet built with Java 8 and running in an Apache Tomcat 8 container. The application and all its dependencies are running within a Docker virtual machine and deployed to a 16-node cluster. The cluster distributes resources using Apache Mesos. With Mesos, the Harmonizome can run on any of the 16 nodes and switch to a new machine if its current node goes down. The Harmonizome database runs on an internal MariaDB server. MariaDB is a drop-in replacement for MySQL. The application communicates with the database through Hibernate object-relational mapping (ORM). An ORM is a framework that maps a tabular schema onto an object paradigm. For example, a single row in the Harmonizome Gene Table is an instance of a Gene class in Java. The search engine uses exact and full-text MariaDB queries to search the database for relevant matches. MariaDB’s natural language search functionality prioritizes the results. We implemented JavaServer Pages (JSP) for most of the user interface. Styling is specified with Less, a Cascading Style Sheets (CSS) pre-processor. We used JavaScript for front-end scripting.
